# Sexual Mixing and HIV Transmission Potential Among Greek Men Who have Sex with Men: Results from SOPHOCLES

**DOI:** 10.1007/s10461-020-03123-6

**Published:** 2021-02-08

**Authors:** Benjamin Bowman, Mina Psichogyiou, Martha Papadopoulou, Vana Sypsa, Aditya Khanna, Dimitrios Paraskevis, Sophocles Chanos, Samuel R. Friedman, Angelos Hatzakis, John Schneider

**Affiliations:** 1 Pritzker School of Medicine, University of Chicago, Chicago, IL USA; 2 First Department of Internal Medicine, National and Kapodistrian University of Athens, Athens, Greece; 3 Department of Hygiene, Epidemiology & Medical Statistics, National and Kapodistrian University of Athens, Athens, Greece; 4 Department of Medicine, Infectious Diseases, University of Chicago, Chicago, IL USA; 5 Athens Checkpoint, Athens, Greece; 6 Institute for Infectious Disease Research, National Development & Research Institutes, New York, NY USA; 7 Department of Population Health, New York University Langone Medical School, New York, NY USA

**Keywords:** HIV, MSM, Greece, Sexual mixing, Network

## Abstract

HIV incidence among men who have sex with men (MSM) in Greece remains unchanged despite effective response to a recent outbreak among people who inject drugs (PWID). Network factors are increasingly understood to drive transmission in epidemics. The primary objective of the study was to characterize MSM in Greece, their sexual behaviors, and sexual network mixing patterns. We investigated the relationship between serostatus, sexual behaviors, and self-reported sex networks in a sample of MSM in Athens, Greece, generated using respondent driven sampling. We estimated mixing coefficients (r) based on survey-generated egonets. Additionally, multiple logistic regression was used to estimate adjusted odds ratios (AOR) and to assess relationships between serostatus, sexual behaviors, and sociodemographic indicators. A sample of 1,520 MSM participants included study respondents (n = 308) and their network members (n = 1,212). Mixing based on serostatus (r = 0.12, σ_r_ = 0.09–0.15) and condomless sex (r = 0.11, σ_r_ = 0.07–0.14) was random. However, mixing based on sex-drug use was highly assortative (r = 0.37, σ_r_ = 0.32–0.42). This study represents the first analysis of Greek MSM sexual networks. Our findings highlight protective behavior in two distinct network typologies. The first typology mixed assortatively based on serostatus and sex-drug use and was less likely to engage in condomless sex. The second typology mixed randomly based on condomless sex but was less likely to engage in sex-drug use. These findings support the potential benefit of HIV prevention program scale-up for this population including but not limited to PrEP.

## Introduction

Of the 628 new HIV infections in Greece in 2017, 292 (46.5%) were among men who have sex with men (MSM), and 8,074 (48.4%) of the 16,669 new HIV diagnoses from 2008–2017 were made in MSM [1]. MSM incidence has remained relatively unchanged in the context of an effective response to an outbreak among people who inject drugs (PWID) [2] and has returned to its pre-outbreak position as the largest percentage of new HIV infections in Greece [1]. Furthermore, some evidence that the PWID epidemic could move into the men who have sex with men (MSM) population has emerged [3].

After the 2010 European MSM Internet Survey (EMIS) provided some behavioral data on MSM in Greece, including sexual practices, HIV testing, antiretroviral therapy, and drug use, information on HIV in MSM in Greece has been improving [4]. Representative data on HIV testing and condom use among MSM is still limited [5]. Other gaps include data on MSM subgroups such as migrants, those who use alcohol and recreational drugs, and those with poor mental health, all of whom may be at higher risk of HIV infection [6].

The higher incidence of HIV among Greek MSM compared to other groups may not be explained by individual-level behaviors alone. It may be attributed in part to pockets of infection [7], sexual network factors, and bridging behaviors between MSM as evident in other contexts [8–10]. Such networks may contribute to the higher incidence rates and provide opportunities for future preventative interventions. For example, higher rates of sexually transmitted infections (STIs) have been found to be related to sexual network mixing patterns such as assortativity and concurrent sex partners [11–14]. Some research has explained disparities in HIV rates by examining sexual network mixing patterns within subgroups [15, 16]. This research demonstrated that higher levels of disassortative mixing (high prevalence groups mixing with low prevalence groups) contributed to disproportionately higher STI incidence.

Since sex practices and network analysis of most vulnerable populations in Greece has focused on PWID [17–21] and not specifically on MSM, despite the majority of cumulative HIV cases being among MSM [19, 22], results from other geographic areas on MSM social and sexual behaviors and networks may be instructive. A study [23] in South Florida, New York City, and Baltimore from 2011 demonstrated that the amount of time MSM lived in an area was associated with sexual behaviors and HIV infection. Known behaviors associated with HIV infection among MSM include sex-drug use [24] and condomless sex (CS) [10]. While these behaviors have been included in previous investigation in Greece [4, 25], they have never been examined among MSM in Greece. Furthermore, network patterns that potentially confer risk, such as disassortative sexual mixing, especially between HIV-positive and HIV-negative MSM, also have not been explored within this population. To that end, an analysis of MSM sexual networks in Greece was conducted characterize associations between network characteristics and HIV serostatus. We hypothesize that mixing between study respondents and sex network members will demonstrate assortative (r ≥ 0.35) [10, 11] mixing for select sex behaviors and serostatus.

## Methods

**Fig. 1:**
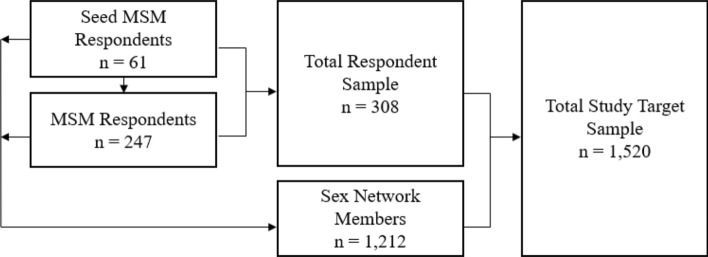
Respondents (n = 308), sex network members (n = 1212), and total participant sample (n = 1520)

Between November 2016 and April 2018, a sample of Greek MSM (n = 308) was recruited in Athens using respondent-driven sampling (RDS) [26]. From that sample, as visualized in Fig. 1, respondents reported sex network members (n = 1212) to generate a total sample of respondents and network members (n = 1520). Computer-assisted interviews were carried out with the respondents by a certified nurse at the partner organization, Checkpoint Athens, a lesbian, gay, bisexual, and transgender testing, prevention, and health information center. Voluntary HIV counseling and testing were conducted in accordance with national regulations and per the standard operating procedures of Checkpoint Athens. Procedures and protocols were approved by institutional review boards at the University of Chicago, the Hellenic Scientific Society for the Study of AIDS and STDs, and the Scientific Council of Laiko General Hospital of Athens. Informed consent was obtained from all respondents.

### Study Participants

Study participants included both respondents who were interviewed and the sex network members about whom they reported. Respondents were eligible if they identified as male, were a resident of the Athens Metropolitan Area, were 18 years of age or older, reported sexual intercourse with a man within the past 12 months, could communicate in either Greek or English, planned to remain in Athens for 12 months, and were willing and able to provide informed consent at the time of the interview. Eligibility was assessed twice, during initial interviews and again during analysis.

### Recruitment

RDS has consistently been used as an effective approach for recruiting and developing population estimates of vulnerable populations such as PWID, sex workers, and MSM [27–30]. Its advantages and shortcomings have been assessed theoretically and empirically [27, 31–33], and this method was selected due to its utility in risk factor identification, network data collection, and implementation of interventions. Previous work emphasized the importance of careful community-informed selection of seeds and iterative rounds of recruitment to thoroughly sample the investigation population [31, 32, 34]. Between November 2016 and April 2018, we followed this strategy to recruit a sample with seeds (n = 61). Seeds who were identified by staff from Laiko General Hospital of Athens and Checkpoint Athens. The recruiter-recruit relationship was not tracked. Seeds were required to meet the above eligibility criteria and demonstrate social connections within the Greek MSM community. Seeds and respondents whom they recruited were remunerated 10 euros (increased to 20 euros after six months to encourage recruitment), distributed five coupons for recruits who met study criteria and had multiple sex partners, and remunerated 5 euros (10 euros as above) for each coupon returned by a recruit who participated in the study. Incentives were increased in May 2017 when recruitment analysis showed an insufficient rate of data collection for the proposed study period to meet levels of precision obtainable from smaller sample sizes in non-RDS studies [31]. We conducted entity resolution to limit the duplication of respondents and sex network partners.

### Survey Instruments

Survey instruments on demographics, behaviors shown to be associated with HIV serostatus, and sexual behavior were adapted from a recent MSM cohort study [35] for face-to-face interviews. Information about age, education, employment status, housing status, marital status, insurance coverage, nationality, sexual practices, PrEP awareness, and drug-use behaviors was collected from respondents in a manner consistent with that used to analyze the HIV outbreak among PWID in Greece in 2012 [20]. Information on respondent condomless sex was solicited through the survey question “When you had anal sex, did you use any condoms?” Respondent sex-drug use was solicited with “When you had sex, did you use drugs or alcohol to make your sexual experience more intense?” [24, 36].

Survey instruments were forward and backward translated from English into Greek and verified by Athens Checkpoint staff.

### Sex Network Assessment

To collect egocentric network data [37], the SOPHOCLES survey instrument adopted established methods that have been used in several other large surveys, including the General Social Survey [38], the National Health and Social Life Survey [39], the National Social Life, Health, and Aging Project [40], and uConnect [41].

Respondents were asked to report behavioral data on individuals with whom they had sex in the 6 months prior to the interview. First, the interviewer elicited a series of sex network members from each respondent. From that list, additional information was obtained on five sex network members. Five was selected given previous work suggesting this is optimal for time and effort in field egocentric network surveys [42]. A series of questions about each sex network member’s demographic attributes and a description of the nature of the relationship followed. Further sex behavior and drug use information on sex network members was also elicited, and referrals for HIV prevention services including testing, counseling, substance use treatment, and pre-exposure prophylaxis (PrEP) were provided.

We focused on two core sex behaviors as our primary outcomes, condomless sex and sex-drug use, given their importance in sexual network mixing and transmission [10] in environments where PrEP is not readily available such as in Athens. For sex network members, condomless sex was measured in response to the question “How often do you believe that this sex partner has anal sex without the use of a condom?" Sex network member sex-drug use was measured in response to the question “How often do you believe that this sex partner uses drugs or alcohol to make sex easier, last longer, or feel better?" These behavioral measures were assessed in frequency terms over the past year and coded as existent if present more than once in the past year.

### HIV Counseling and Testing

Interviews, testing, and counseling were all carried out at the Athens Checkpoint office with linkage to care at Laiko General Hospital of Athens as appropriate. All respondents were initially tested for HIV with a fingerstick, using the INSTI HIV-1/HIV-2 Rapid Antibody Test. In the case of positive tests, follow-up blood testing with Genscreen™ ULTRA HIV Ag-Ab, ARCHITECT HIV Ag/Ab Combo, and Bio-rad Western Blot testing was used to confirm serostatus. Checkpoint staff conducted pre- and post-test counseling with all respondents.

### Respondent-Level Analyses

We conducted three multiple logistic regressions to determine the relationship between serostatus, the sex behaviors just described, and sociodemographic characteristics as in this study [43]. The first regression assessed the effect of variables such as sex behaviors and sociodemographic characteristics on HIV serostatus. In the second regression, the outcome was sex-drug use and in the third condomless sex. In each model, we adjusted for age, education status, nationality, and relationship status. Seeds were included in analysis. RDS weights were applied to all models using the Giles Sequential Sampling approach [44]. Models were also run without RDS weights as in similar work [45].

### Mixing Analysis

Assortativity coefficients (r) [46] were calculated to describe the sexual mixing patterns (i.e. homophilic or “like-with-like”) in our sample. For HIV status, for example, by tabulating the proportions of the two types (HIV-positive with HIV-positive and HIV-negative with HIV-negative) of seroconcordant dyads and the two types of serodiscordant dyads (HIV-positive with HIV-negative and HIV-negative with HIV-positive), a two-by-two mixing matrix of the four types of ties above was constructed. Using a similar technique, assortativity coefficients with 95% confidence intervals (CI) were computed for sex-drug use and condomless sex. An assortativity coefficient of r = 1 indicates complete assortative mixing, where all ties would be seroconcordant. Coefficient of r = − 1 would indicate total disassortative mixing, where all ties would be serodiscordant. When r = 0, this indicates random mixing [47]. Based on previous work, coefficients of 0.35 or larger are generally characterized as assortative, 0.26 to 0.34 as moderately assortative, and 0.15 to 0.25 as minimally assortative [10, 11].

## Results

The sample included a total network (n = 1,520) generated from 61 seeds and included respondents (n = 308) and sex network members (n = 1212) as demonstrated in Fig. 1. The RDS seeds generated the full respondent sample with non-zero networks averaging 17.1 seed respondents and a maximum number of 11 waves. Seed productivity demonstrated a wide range (0–107) with 35 non-productive seeds (57.3%).

### Respondent and Sex Network Member Characteristics

Attributes of respondents and sex network members are depicted in Table 1. The prevalence of HIV in respondents (8.4%) and sex network members as known by respondents (10.4%) were similar. HIV prevalence of sex network members reported by seropositive respondents (20.0%) was double that (9.2%) reported by seronegative respondents about their sex network members. HIV PrEP awareness among respondents was 73.7%. A majority of respondents (87.0%) and sex network members (83.2%) were Greek nationals, and a majority of respondents (60.0%) and sex network members (70.5%) were employed.

**Table 1:** Respondent (n = 308) and sex network member (n = 1212) attribute

Attributes	Respondents N (%)	Sex network members N (%)
Age		
< 20	9 (3.0)	14 (1.2)
20–24	99 (32.1)	239 (20.3)
25–34	123 (40.0)	552 (50.0)
35–45	57 (18.5)	307 (26.1)
46 +	20 (6.5)	64 (5.4)
Education^a^		
Junior high school or minor vocational schools, primary school, less than primary school	3 (1.0)	–
Senior high school, vocational high school	72 (23.4)	–
Private vocational schools and colleges	49 (15.4)	–
Technological educational institutes, Universities, Military academies	133 (43.2)	–
Master of arts/science, doctoral degree	51 (16.6)	–
Employed	185 (60.0)	854 (70.5)
Gender		
Male	308 (100)	1,193 (98.4)
Female	0 (0)	17 (1.4)
Trans	0 (0)	2 (0.2)
HIV status		
HIV+	26 (8.4)	95 (10.4)^b^
HIV−	282 (91.6)	822 (89.6)^b^
Nationality		
Greek	268 (87.0)	1,008 (83.2)
Other^c^	40 (13.0)	204 (16.8)
Risk network member		
At least 1 sexual partner is HIV positive	47 (15.3)	110 (9.1)
No sexual partner is HIV positive	261 (84.7)	1,102 (90.9)
PrEP awareness		
Yes	227 (73.7)	–
No	81 (26.3)	–
PrEP use		
Yes	–	31 (2.6)
No	–	1154 (95.2)

^a^Education status not collected for sexual partners

^b^From among those who indicated that respondent knows sex network members’ HIV status

^c^Includes Albanian, Russian, Iranian, Afghan, Kurdish, Pakistani, Arab, African, other European, other

The distribution of sexual behavior characteristics for respondents and respondents' perceptions of their sex network members' behavior is depicted in Table 2. Sex-drug use for respondents (45.6%) and respondents' perceptions of the sex-drug use of their sex network members (54.4%) were more similar than condomless sex for respondents (17.6%) and respondents' perceptions of the condomless sex of their sex network members (62.7%). The distribution of sexual behavior characteristics stratified by respondent HIV status is also depicted in Table 2. Again, sex-drug use showed less difference. Similar sex-drug use reported by seropositive (45.4%) and seronegative (45.6%) respondents was about 10% lower than seropositive (56.7%) and seronegative (54.1%) respondents' perceptions of the sex-drug use of their sex network members. However, the condomless sex reported by seropositive respondents (30.0%) was nearly twice that of seronegative respondents (16.2%). Furthermore, respondents reported significantly higher condomless sex by their sex network members regardless of whether the respondent was seropositive (58.7%) or seronegative (61.3%).

**Table 2:** Distribution of sexual behaviors and characteristics for respondents (n = 308) and sex network members (n = 1,212) by tested HIV status of respondents

	Respondents			Sex network members		
	All	HIV+ ^a^ N (%)	HIV− N (%)	All	HIV+ N (%)	HIV− N (%)
*N* total	308	26 (8.4)	282 (91.6)	1212	110 (9.07)	1102 (90.9)
Sex drug^b^	104 (45.6)	10 (45.4)	94 (45.6)	571 (54.4)	59 (56.7)	512 (54.1)
Condomless sex^b^	36 (17.6)	6 (30.0)	30 (16.2)	635 (62.7)	54 (58.7)	581 (63.1)
HIV seropositive^b,c^				95 (10.4)	19 (20.0)	76 (9.2)

^a^"HIV+ " and "HIV− " refers to the serostatus of respondents

^b^Percentages exclude missing cases

^c^HIV seropositive refers to status of network members as reported by respondent (excluding missing cases)

### Respondent-Level Regression Results

In regression analysis with adjusted odds ratios (AOR) depicted in Table 3, two sexual behaviors demonstrated statistical significance. Respondents who reported sex-drug use were significantly less likely to report condomless sex (AOR 0.47; 95% CI 0.27, 0.80) and respondents who reported condomless sex were significantly less likely to report sex-drug use (AOR 0.49; 95% CI 0.27, 0.80).

**Table 3:** Saturated multiple logistic regression analysis^a^ model of respondent behaviors and attributes on HIV status, sex-drug use, condomless anal intercourse, and group sex among Men who have Sex with Men in Athens (n = 308)

Characteristic	HIV status	Sex-drug use	Condomless sex
	AOR (95% CI)	AOR (95% CI)	AOR (95% CI)
Respondent behaviors			
Sex-drug use	0.97 (0.38, 2.46)	…	0.49* (0.27, 0.80)
Condomless sex	0.84 (0.34, 2.05)	0.47** (0.27, 0.80)	…
Respondent characteristics			
Age	1.10*** (1.05, 1.15)	1.03 (0.99, 1.06)	1.02 (0.99, 1.05)
PrEP awareness	0.92 (0.30, 2.82)	1.38 (0.70, 2.70)	0.95 (0.53, 1.72)
Sex network size	0.99 (0.98, 1.01)	1.00 (1.00, 1.00)	1.00 (0.99, 1.00)
Education status	0.83 (0.54, 1.27)	1.00 (0.77, 1.29)	1.26 (0.98, 1.60)
In a relationship^b^	1.44 (0.56, 3.70)	0.55 (0.29, 1.04)	1.34 (0.77, 2.33)
Non-Greek nationality	0.50 (0.15, 1.61)	0.37* (0.18, 0.79)	0.30** (0.14, 0.64)
Risk network membership^c^	…^d^	2.49 (0.93, 6.62)	1.03 (0.39, 2.72)

*AOR* adjusted odds ratio, *CI* confidence interval

^a^The model is an analysis of the association between respondent sexual behaviors and characteristics. The model controls for age, self-reported sexual orientation, employment status, relationship status, HIV status, education, PrEP awareness, and size of sexual network

^b^Self-reported as married or having a boyfriend

^c^At least 1 sex network member is HIV positive

^d^Excluded as perfect predictor of HIV status

^*^p < 0.05, **p < 0.01, ***p < 0.001

Other statistically significant associations included that older respondents were more likely to be HIV-positive (AOR per annum 1.10; 95% CI 1.05, 1.15). Non-Greek respondents were less likely to report condomless sex (AOR 0.30; 95% CI (0.14, 0.64).

### Sex Tie Characteristics

Characteristics of ties between respondents and sex network members are shown in Table 4. A minority of the ties measured (17.9%) occurred in the context of a relationship such as husband or boyfriend. More than two-thirds (66.9%) of ties were established via the internet or telephone. Additionally, one in ten (10.9%) of respondents reported that at least one of their last five sex network members in the past 6 months had sexual contact with another identified member, creating concurrent ties and sex network triads.

**Table 4:** Respondent (n = 308) and sex network member (n = 1212) tie characteristics

Tie characteristics	Dyads (%)
Relationship types	
Husband	30 (2.5)
Boyfriend or girlfriend	187 (15.4)
Sexual partner	575 (47.4)
Client for sex work	14 (1.2)
Sex worker	3 (0.3)
Casual sex partner who I do not really know	100 (8.3)
Other^a^	297 (24.5)
Relationship establishment	
Mutual friends	167 (13.8)
Internet or telephone	811 (66.9)
Party or place of group drug use	34 (2.8)
Gay event	44 (3.6)
Other^b^	156 (12.9)
Sexual mixing of sexual partners with other sexual partners^c^	
At least one sexual partner with other sexual partner	234 (10.9)
No connection between past sexual partners	1753 (81.8)

^a^Includes one-night stand, friend, friend with benefits, other

^b^Includes work, bar, sex party, school, Checkpoint Athens, concert, sauna, conference, roadside, bus station, metro station, other

^c^Indicated by respondent from among last 5 sexual partners in last 6 months

### Network Mixing

**Fig. 2:**
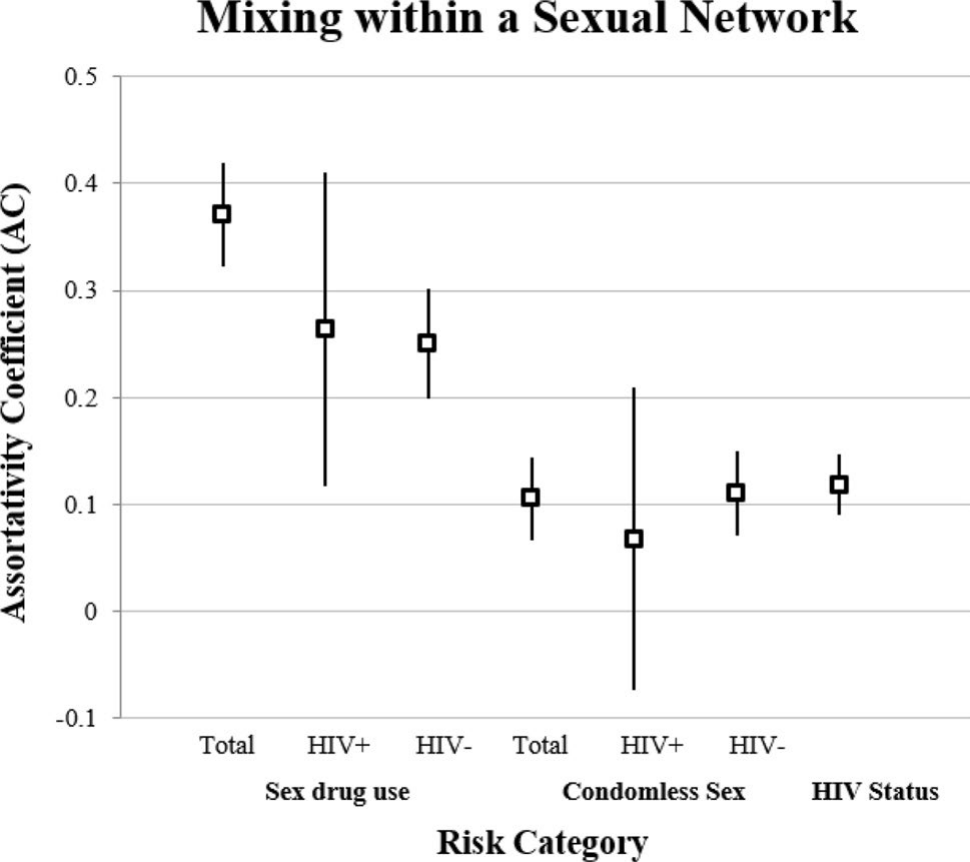
Mixing within sexual networks by sexual behavior and HIV status (n = 1550). Assortativity coefficients (ACs)^a^ for two behaviors—sex-drug use and condomless sex—and HIV status of MSM respondents and sex network members are depicted here. Nodes and error bars within each behavior category indicate the AC for all dyads in the sample. ACs are also stratified by HIV status of respondents. An AC of 1 would indicate perfectly assortative mixing, e.g., respondents who practice a behavior only have sex with those who also practice that behavior, while respondents who do not practice the behavior only have sex with those who also do not practice the behavior. ^a^The Assortativity Coefficient (AC) is calculated from the mixing matrix—the proportion of total ties in a cross-tabulation of ties between people who do and do not engage in a sexual behavior

Our mixing analysis of sexual behaviors (sex-drug use and condomless sex) is depicted in Fig. 2 and further stratified by HIV status.

Mixing of study participants based on sex-drug use was assortative (like with like) (r = 0.37, σ_r_ = 0.32–0.42). When stratified by tested HIV status of respondents, mixing based on sex-drug use in the HIV-positive stratum was moderately assortative (r = 0.26, σ_r_ = 0.12–0.41). Similarly, mixing based on sex-drug use behavior in the HIV-negative stratum was also moderately assortative (r = 0.25, σ_r_ = 0.20–0.30).

Mixing with regards to condomless sex and HIV status did not display assortative mixing. Respondents and sex network members mixed randomly based on condomless sex (r = 0.11, σ_r_ = 0.07–0.14) and positive HIV status (r = 0.12, σ_r_ = 0.09–0.15). In our study then, except for sex-drug use, the sexual behavior of respondents had no tendency to be similar to that of their self-reported sex network members even when serostatus was considered. Weighted and unweighted results did not differ.

## Discussion

While attention has been devoted to sex behaviors [4] and drug-use networks [19, 20] in Greece, most HIV network epidemiology in Greece has focused on PWID [18, 20, 21, 48, 49]. Our study represents the first network analysis that investigated the role of networks with regards to sexual behaviors and HIV serostatus in MSM in Greece.

We have two major findings related to prevention of onward transmission of HIV in this context. The first is a network typology of MSM demonstrating assortative mixing based on sex-drug use who seem to have adopted two protective behaviors (serosorting and sex with condoms) and who have an unclear awareness of PrEP. The second is a network typology of MSM engaging in condomless sex who seem to have adopted one protective behavior (avoiding partners who engage in sex-drug use) and also have an unclear awareness of PrEP. Both represent intervention opportunities.

Participants demonstrated assortative (r ≥ 0.35) [25] mixing for only one sexual behavior: sex-drug use. This suggests that Greek MSM who engage in sex-drug use behavior perceive their sex network members as having similar sex-drug use behavior.. Furthermore, seropositive Greek MSM who also engage in sex-drug use tend to do so with other seropositive MSM. Seronegative Greek MSM similarly self-segregate when engaging in sex-drug use. The observed assortative sexual mixing pattern for sex-drug use is consistent with previous findings and is an example of serosorting—a behavior that limits sexual activity to partners of the same serostatus and is thought to be protective in some contexts [15, 16, 50, 51]. The finding in regression analysis that those who use drugs to enhance sex have adopted a second protective sexual behavior (avoiding condomless sex) suggests this group could be an effective target [52] of safe drug and condom use interventions. The same phenomenon could occur with PrEP use considering this group's limited PrEP awareness (AOR 1.38; 95% CI 0.70, 2.70). Thus, the first network typology still seems amenable to promotion of condom and PrEP use in the context of sex-drug use.

Contrary to our hypothesis and what has been observed in other MSM populations [10], no sex network sorting on other behaviors was apparent. Network member preference for partners with similar behavior profiles for condomless sex and HIV status was found to be nearly random (r ≤ 0.15) [9] regardless of stratification by serostatus. These heterogenous results are surprising because sex ties have been shown previously to be based on shared sexual behaviors [10, 11]. While evidence of preference with regards to only one sex behavior is inconsistent with more comprehensive strategic mixing found previously, it does not suggest random mixing as the driving factor behind the unchanged HIV incidence in Greek MSM. Other research has demonstrated levels of serosorting to be unrelated to HIV incidence disparities between sub-populations [53, 54]. Additionally, the random mixing based on serostatus and condomless sex could be viewed as an interventional target. Finally, since our analysis did not investigate the incidence rates of other STIs, we cannot examine how mixing contributed to their incidence.

Although the second network typology demonstrated neither serosorting nor sorting based on condom use in mixing analysis, those respondents did show a protective preference for partners with no sex-drug use in regression analysis. Having adopted this protective behavior, the second typology also represents an interventional target. Such interventions should take note of the protective preference and its implication on behavior propagation.

Social learning and differential association theories [55, 56] assert that sex behaviors and the conceptualizations for them propagate through social and sexual networks. Network members demonstrate and normalize specific sex behavior, reshaping MSM perceptions of these behaviors [57–59]. Research has shown in various contexts that individual perception of network members can induce individual sexual and substance use behaviors [10, 60–62]. In light of these theories, random mixing on condomless sex has implications on the propagation of this behavior and through Greek MSM sexual networks, especially as evidence of decreasing condom use with PrEP rollout emerges [63] in some locations. For example, in a state of random mixing such as observed here, sexual partners of different condom use preferences are more likely to encounter each other. Conversely, network members mixing assortatively would more rarely encounter partners with different preferences. The higher frequency of encounters between partners with different condom use preferences presents more opportunities for perception change and behavior propagation. Such interventions should take note of the protective preference and its implication on behavior propagation.

While disassortativity for condomless sex has been observed in other MSM networks globally, cautious comparison of these results is still necessary due to the vastly different populations and settings. Regardless, for the second network typology, stakeholders could look to primary prevention efforts such as PrEP provision, condom use promotion, and increasing the awareness of the HIV status of sexual partners as well as secondary prevention efforts such as programs that engage with HIV-positive MSM to prevent onward transmission. Evidence-based interventions more targeted to specific network characteristics [64, 65] are still developing and require further investigation.

As an update of the demographics of MSM in Greece, the prevalence of HIV in the study sample as reported above was consistent with that reported among Greek MSM by the European Center for Disease Control (ECDC) in 2014 (6). However, sex-drug use was significantly higher for respondents (45.6%) and sex network members (54.4%) in our sample than regional percentages (6.6%) reported in EMIS in 2010 [4]. Condomless sex was slightly lower.

The continuing effort to illuminate the sex networks and transmission of HIV between Greek MSM must go beyond the mixing of high-risk and low-risk individuals. Social ties have been shown to be critical to disease transmission [10, 66]. Due to collectivist cultural and religious influences [67], they may be even more significant in Greece. Therefore, longitudinal social *and* sex network analysis should be pursued to unravel the nexus of sexual behavior profiles, assortativity, social influence, behavior norms and normalization, and network behavior propagation to construct better individual and network interventions.

### Limitations

Several potential limitations affect this study. The aftermath of the 2008 economic crisis on Greece is still playing out. Rigorous research has shown its effects on the spread of HIV among PWID [17–20]. The possibility of residual influences on the MSM population cannot be ignored, especially as phylogenetic evidence of spillover of the PWID epidemic into the MSM population continues to emerge [68].

Despite the great need for sampling and inference methods to investigate hard-to-reach groups, uncertainty regarding how well RDS generates representative samples and unbiased analysis still faces investigators [31]. Even the uncertain ability to detect bias in RDS impels the reader to consider that our CIs could be too narrow and that inferences drawn from our sample may not be applicable to a wider population. Additionally, data analysis and anecdotal investigation could not elucidate why 35 seeds were non-productive.

As is typical of egocentric network analysis, all data were self-reported by respondents, and thus are susceptible to projection bias [69] and pluralistic ignorance [70]. This limitation could lead to overreporting of shared characteristics and thus falsely elevated assortativity. Respondents have also been shown to under-report their own stigmatized behavior and over-report their partners' behaviors [71–73], which would falsely depress assortativity. Another limitation is the potential overlap between respondents and sex network partners. Finally, the low number of HIV-positive participants in the sample lead to weak variability and increased the standard error for measurements related to HIV-positive participants, possibly reducing the power to identify associations.

## Conclusion

This study represents the first network analysis of the sexual mixing patterns of Greek MSM. Our two major findings relate to protective sexual behavior among MSM in Greece. The first is a network typology of serosorting MSM, engaging in assortative mixing based on sex-drug use, who are more likely to engage in sex with condoms. The second is a network typology of MSM, who despite being less likely to engage in sex-drug use, mix randomly based on condomless sex and are thus susceptible to the propagation of that sexual behavior seen elsewhere. Both represent interventional opportunities to reduce transmission, slow the spread of HIV, and facilitate prevention. The effectiveness of PrEP in these contexts should be explored.
